# Anakoinosis: Correcting Aberrant Homeostasis of Cancer Tissue—Going Beyond Apoptosis Induction

**DOI:** 10.3389/fonc.2019.01408

**Published:** 2019-12-20

**Authors:** Daniel Heudobler, Florian Lüke, Martin Vogelhuber, Sebastian Klobuch, Tobias Pukrop, Wolfgang Herr, Christopher Gerner, Pan Pantziarka, Lina Ghibelli, Albrecht Reichle

**Affiliations:** ^1^Department of Internal Medicine III, Hematology and Oncology, University Hospital Regensburg, Regensburg, Germany; ^2^Institut for Analytical Chemistry, Faculty Chemistry, University Vienna, Vienna, Austria; ^3^The George Pantziarka TP53 Trust, London, United Kingdom; ^4^Anticancer Fund, Brussels, Belgium; ^5^Department Biology, Università di Roma Tor Vergata, Rome, Italy

**Keywords:** anakoinosis, communicative reprogramming, master modifiers, systems biology, metastatic tumors, reprogramming information flux

## Abstract

The current approach to systemic therapy for metastatic cancer is aimed predominantly at inducing apoptosis of cancer cells by blocking tumor-promoting signaling pathways or by eradicating cell compartments within the tumor. In contrast, a systems view of therapy primarily considers the communication protocols that exist at multiple levels within the tumor complex, and the role of key regulators of such systems. Such regulators may have far-reaching influence on tumor response to therapy and therefore patient survival. This implies that neoplasia may be considered as a cell non-autonomous disease. The multi-scale activity ranges from intra-tumor cell compartments, to the tumor, to the tumor-harboring organ to the organism. In contrast to molecularly targeted therapies, a systems approach that identifies the complex communications networks driving tumor growth offers the prospect of disrupting or “normalizing” such aberrant communicative behaviors and therefore attenuating tumor growth. Communicative reprogramming, a treatment strategy referred to as anakoinosis, requires novel therapeutic instruments, so-called master modifiers to deliver concerted tumor growth-attenuating action. The diversity of biological outcomes following pro-anakoinotic tumor therapy, such as differentiation, trans-differentiation, control of tumor-associated inflammation, etc. demonstrates that long-term tumor control may occur in multiple forms, inducing even continuous complete remission. Accordingly, pro-anakoinotic therapies dramatically extend the repertoire for achieving tumor control and may activate apoptosis pathways for controlling resistant metastatic tumor disease and hematologic neoplasia.

## Introduction

The dominant paradigm for systemic therapy of metastatic tumors is based on molecularly targeted therapies (1). Although revolutionizing cancer therapy, several characteristic obstacles may contribute to therapeutic failure. Frequently, the primary aim of such therapies is induction of apoptosis—an aim which is frequently only partially successful, or which is effective only for short time-intervals. Tumors respond to therapy heterogeneously, due to intra-tumor genetic heterogeneity. Commonly pre-therapeutic tumor evaluation does not integrate data on the tumor's phenotype. Diagnostics do not answer biologically pertinent questions: what are the phenotypes determining organ-tropism? How is invasion physically constituted? Do the hallmarks of cancer vary significantly between primary site and metastases, for example immune escape? (2, 3). Further, the selection of sequential therapies do not sufficiently consider the influence of preceding treatments on tumor evolution—which may influence outcome in a long run (4). Additionally, the compromising of the whole organism by the tumor, treatment side effects due to maximum tolerable doses, and finally, comorbidities, social and psychological circumstances can decisively determine therapy outcome.

The systems view can be in the truest sense of the word “created” by bottom-up considerations resulting in knowledge-based mathematical models (5). Alternatively, systems may be studied by hypothesis-driven top-down approaches, including regulatory active drug combinations (6, 7). Those schedules induce phenotypical tumor changes facilitating the description of the therapeutically accessible search space for therapeutic options. This huge space for therapeutic possibilities optionally expands depending on the multi-level availability of regulatory accessible, communicatively linked systems functions coordinating heterologous cell types, exemplarily among cells in the tumor tissue, and tumor-organ/ -organism interactions (8–11).

The methodology of reprogramming tumor systems reveals the intercellular communications “protocols” that connect multiple system components, including heterogeneous cell types, even in the dysregulated states that make up the hallmarks of cancer. Based on a biomodulatory approach, this reprogramming strategy addresses, systematically, several of the impediments to molecularly targeted therapies, as has been shown in therapeutically relevant ways. This novel approach, which has been termed “anakoinosis,” is predicated on the administration of such master modifiers in metastatic and refractory disease. The term anakoinosis is based on a Greek word (α*νακo*ι*νωσεις*) which means shared communication or information and is a fitting description of this new treatment paradigm.

The review outlines the communication theoretical aspects of pro-anakoinotic tumor therapy; it describes tumors as a design space from a biological viewpoint; as well as presenting the clinical approach of pro-anakoinotic tumor therapy. Finally, the review provides perspectives for the use of anakoinosis-inducing approaches and the implications of anakoinosis for toxicology.

## Tumor as a Therapeutically Accessible Design Space: The Biologic View

**Figure 1:**
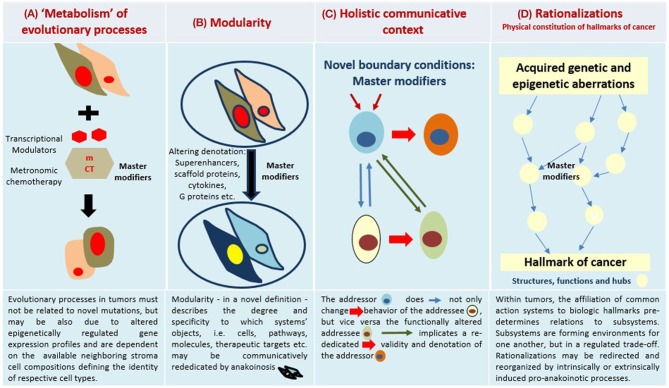
Four major communication tools for inducing anakoinosis: Changing flux of information. Concertedly, oncogenic events and “recessively” developing disease traits constitute the tumor phenotype, which is communication-technically mediated by the tumor's “background knowledge.” Clinical data on pro-anakoinotic therapy approaches indicate successful therapeutic modeling of homeostatic processes, including distant organ sites. Pro-anakoinotic therapies with their regulatory activity profile may sideline classic tumor-promoting pathways or cells with oncogenic load by activating alternative communication flux. Thus, pro-apoptotic pathways may be re-activated, or tumors may be kept in check. The observed communication-technical functional reset of the tumors' heterologous cell compartments reveals a set of general communication rules, which are accessible for a broad diversity of biomodulatory interventions. Biologic tumor features, which are communication-technically accessible, are severely dysregulated transcriptional programs, homeostatic pathways, immune responses and down-regulated tumor suppressor genes, respectively. **(A)** The sum of extrinsically, i.e., therapeutically, and intrinsically inducible evolutionary processes within the tumor environment (tumor stroma, hosting organ, distant organ sites). **(B)** Modular events: Changing validity (availability on demand at distinct time points) and denotation (current functional impact at a distinct systems stage of systems objects. **(C)** Communicative interactions of the tumor with tumor hosting organ and the organism for generating novel functions, structures and hubs, thereby defining cell identity. **(D)** Hallmarks of cancer are differentially physically realized and constitute normative notions; are to some degree histology- and genotype-independent; may be re-directed and reorganized by anakoinosis.

To fully appreciate the conceptual breakthrough implicit in anticancer anakoinotic treatment one must consider that exploitation of epigenetic and homeostatic notions requires us to “exit” from the cell level and to consider the **tissues as functional units**. Even though it is common knowledge that tissues are not merely a simple agglomeration of cells, but can be seen as societies where communicative signals are continuously exchanged thus determining cells' behavior, the implications are often disregarded (12). The notion that the transcription pattern of each cell is mainly determined by the signals coming from the tissue (i.e., non-cell autonomous), is vitally important. This notion suggests that therapeutic targets may shift from cells (as occurs in classical apoptosis-inducing anticancer treatments) to tissues. This latter is a less clearly identifiable target, because what should be pursued is not the destruction of the wrong object, but the change in the flux of information (Figure 1). To this purpose notions regarding homeostatic rules, learnt from the developmental biology, must be elaborated to gain a picture that may be employed in therapeutic anticancer prospective (13–15).

### Tumor Tissue, a Communicative Unit: Inducing Change in the Flux of Information

A major concern in considering the tumor microenvironment as the preferred target for anticancer therapies is that the presence of mutated cells may render any attempt at cancer-tissue reconditioning a transient phenomenon, because eventually the oncogene-determined phenotype will re-emerge. However, a significant body of research unequivocally shows that the malignant features of cancer cells persist only within the cancer tissue and are lost upon re-localization in normal environments. This is despite oncogene mutations: when outside tumors, cancer cells differentiate or die because the correct homeostatic pathways of the recipient tissues recognize them as anomalous (16). These notions imply that correcting the cancer microenvironment would create a condition, within the tissue itself, where cancer cells, whatever the oncogenic mutations they bear, are induced to die or differentiate by endogenously driven mechanisms, mimicking those of spontaneous tumor regression reported e.g., in some skin cancers (17).

Furthermore, it is important to consider that it is practically impossible to fully describe a cell or tissue in molecular terms—in living beings change is constant and case-by-case variability an inherent property (9). In such a scenario, it is no simple task to find the proper molecular targets. The continuous exchange of molecular (lipid and protein ligands) and physical (e.g., matrix stiffness) messages taking place within a tissue implies continuous adjustments in cells' epigenome, proteome and lipidome, producing an ever-changing molecular pattern, whose turnover is especially rapid in conditions of stress or damage, such as those occurring in cancer (9, 18–20). This makes the targeted therapy paradigm very much at risk of being nullified by the abundant number of alternatives cells can choose from, at any moment. Instead, actions aimed at correcting the flux of information, that is, targeting not the *object*, but the *process*, are not in the need of thorough moment-by-moment molecular description, thereby bypassing the obstacle of this kind of biological “uncertainty principle” (9) (Figure 1).

This logic leads us to suggest that cancer tissues should not be viewed in reductionist terms, that considerations of aberrant homeostasis are important, and that discerning communicative dysfunction must be a prime consideration in thinking about cancers. As in developmental biology a shift in paradigm is called for Heudobler et al. (10).

### Cancer Cell Identity Is Determined by Genetic as Well as by Tissue-Driven Gene Expression Modulation

Mutations in oncogenes and tumor suppressor genes are still considered the most important feature characterizing cancer cells. In most clinical cases of cancer, and in translational models, mutations in genes controlling cell proliferation confer the ability to push the cell cycle without stimulation from the tissue, inducing uncontrolled proliferation. The consequent replicative stress, in normal cellular and tissue contexts, is typically controlled by intrinsic cell and tissue anticancer defenses. Tumor suppressor genes also induce cells with mutated oncogenes toward apoptosis or senescence (oncogene-induced apoptosis or oncogene-induced senescence), thus eliminating dangerous cells (21). Tumor suppressor genes are typically silenced in cancer, and therefore cells in replicative stress are able to survive and give rise to populations of cancer clones. Therefore, the tumor cell's genotype does not *exclusively* determine tumor behavior, but also non-autonomous regulated gene expression patterns, which control the on-off switch of tumor suppressor expression (22). Thus, the identity of cancer cells is also determined by non-cancer cell autonomous, communicatively mediated mechanisms; in the same guise, these mechanisms determine the identity of a tissue-specific cell in multicellular organisms. The phenotypes of glioblastoma cells, for example, may be reversibly shaped by microenvironmental events (23). In fact, re-establishing tumor suppressor expression can overcome continuous proliferation and stop cancer growth (24).

In addition to the classical tumor suppressor genes controlling cell proliferation by eliminating mutant cells, data is emerging that tissue-coordinated defenses allow cells bearing mutated oncogenes to survive and function within tissues. This occurs via signals controlled by cell-polarity-controlling genes (25) and is a mean of sparing cells that preserve tissue function. This is especially critical in stressed tissues or in tissues from aging organisms, tissues bearing cells subject to frequent mutations from environmental or endogenous causes, respectively. This signaling network allows cells with oncogene expression to survive, possibly by interfering with the proliferative signals downstream of oncoprotein synthesis, suggesting that activated oncogenes may perform additional, non-cancer related functions. For example, a single-cell DNA sequencing study performed on specimens from blepharo-plastic surgery in elderly individuals with no clinical tissue alterations showed that these tissues, histologically normal, bore a burden of oncogene mutations in terms of number and type of genes similar to those found in cancer specimens. The spatial distribution of such cells suggested that the mutated oncogenes provided a selective advantage over their non-mutated counterparts, indicating they were expressed without causing cancer (26). An important study suggests a possible mechanism for this apparently paradoxical finding: it has been shown that conditional expression of oncogenic MYC in normal breast cells promotes uncontrolled replication in traditional cell cultures, but not in cells cultured in 3D conditions with a proper extracellular matrix analogous to regular breast acinus. Interestingly, matrix digestion induced MYC-expressing cells to exit from the acinus and undergo oncogene-induced apoptosis (27, 28). Such studies have very important implications, showing that at least two levels of anticancer defenses exist in epithelial tissues, indicating that a tissue-level defense, acting via control of cell polarity, exists and acts upstream of the classical anti-proliferative tumor suppressor genes of the RB and TP53 families (29).

These studies indicate that oncogene mutation is not sufficient to induce cancer. However, is oncogene mutation necessary? Theoretically, continuous activation of the MAP kinase pathway, an event that characterizes cancer cells mutated in the Raf/Ras families, may also be achieved by forced expression of one or more *wild-type* genes. Clinically, this is sometimes found in human virally induced carcinogenesis, which are typically characterized by a low oncogene mutation load (30). The highly organized attack that oncoviruses direct at infected cells indeed includes transactivation of oncogenes, which thus are continuously activated even in the absence of a direct mutational event.

Therefore, oncogene mutation in cancer is neither necessary nor sufficient, even though it clearly facilitates tumor genesis and progression, although in practice clinical cancers without oncogene mutations are practically never found. However, this principle clearly states that cells bearing mutated oncogenes may be kept at bay by tissue-level defenses.

Is this applicable to cells that already express a malignant phenotype in a tissue with aberrant cancer homeostasis? Clearly, the abundant literature showing that cancer cells placed in a healthy environment either die or are normalized indicates that this is possible. Probably harder is restoring correct homeostasis in a tissue that has already lost normal homeostasis. However, the clinical achievements of anakoinosis therapies to date show that it is a very promising approach (10). Therefore, “evolving” metastatic disease in a clinically meaningful way is of important therapeutic impact. Differentiation induction, transdifferentiation, biological memory, mobilization of alternative apoptotic pathways via non-oncogene addicted targets may significantly extend the impact of non-curative care to achieve continuous complete remission (9–11, 31–33).

### Multiscale Modeling of Communication Processes in Tumor Systems

It is important to mention the multiscale modeling of communication processes in tumor systems, and how oncogenic events alter homeostatically well-balanced cell systems. In so doing, such alterations promote additional oncogene-unrelated targets, finally characterizing the tumor's phenotype. Oncogenic-events drive tumor-associated “distorted” evolutionary processes, but vice versa, only the specific communicatively developing microenvironmental response attributes to the context-mediated biologic validity and denotation of respective oncogenic events (Table 1) (9, 34, 35). Thereby, the tumor microenvironment influences pharmacodynamics, regulates the regrowth capacity of surviving tumor cells, and mediates drug resistance (36).

**Table 1:** Glossary: explanation of communication associated terms.

**Communication associated terms**	**Explanations**
Anakoinosis	Anakoinosis is a novel paradigm for cancer treatment based on therapeutic modulation of biological communications processes and aims at prioritizing alternative pathways for apoptosis induction, normalizing activity of dysregulated homeostatic pathways, at up-regulating non-mutated tumor suppressor genes, attenuation of stroma-mediated support for tumor growth, and at modulating cancer checkpoints. The methodology of reprogramming tumor systems reveals the intercellular communications protocols that connect multiple system components, including heterogeneous cell types, even in the dysregulated states that make up the hallmarks of cancer. This reprogramming strategy addresses, systematically, several of the impediments to molecularly targeted therapies.
Reverse anakoinosis	Induction of oncogenic events via concerted activity of non-oncogenic, but pro-anakoinotic agents.
Master modifiers	The diversified activity profiles of **master modifier combinations** in tumor tissue, differentiation induction, transdifferentiation, control of tumor-associated, growth-promoting inflammation, immunologic control etc., indicate the broad therapeutic impact of transcriptional modulators, nuclear receptor agonists and antagonists, metronomic low-dose chemotherapy, cyclooxygenase-2 inhibitors, IMiDs, arsenic trioxide, liposomal encapsulated small oligonucleotide encoding small activating RNAs, repurposed targeted therapies for non-oncogene addicts, vaccines (immune), checkpoint inhibitors etc. In contrast, master regulators are on tissue site available regulatory structures.
Metronomic low-dose tumor therapy	Metronomic tumor therapy may be defined as the frequent administration of (repurposed) drugs at doses significantly below the maximum tolerated dose with no prolonged drug-free breaks, or as the minimum biologically effective dose of an agent given as a continuous dosing regimen with no prolonged drug-free breaks still leading to antitumor activity.
Validity and denotation	Validity of systems objects, functions and hubs: Availability on demand at distinct systems stages; denotation: Current functional impact at a distinct systems stage, e.g., of potentially tumor-promoting pathways. In the bio-world, presence and functioning of an object (e.g., an enzyme), respectively.
Physical constitution of hallmarks	Describe the physical organization of tumor-associated normative notions (e.g., hallmarks of cancer); are to some degree histology- and genotype-independent; may be re-directed and reorganized by anakoinosis.
Evolutionary processes in tumors	The sum of **extrinsically**, i.e., therapeutically, and **intrinsically** inducible **evolutionary processes** within the tumor environment (tumor stroma, hosting organ, distant organ sites).
Background knowledge	Cell compartments and tissue systems, organs and organisms have the capability to respond with their available communication tools to oncogenic challenges, thereby activating various response patterns, i.e., acute and chronic evolutionary processes (tumor disease), repair mechanisms (reconstitution ad integrum or defect healing), apoptosis or death of the organism. Contently, the sum of possible response patterns represents the respective systems' “background knowledge.” This communication technical tool supplies robustness, evolvability and repair within cellular systems following endogenous or therapeutic activation.
Tumor checkpoint	A autoregulated module comprising master regulator proteins maintaining tumor cell state.
Non-oncogene addiction	Tumors may become dependent on recessively developing dysregulated master regulators. Dysregulated master regulators and checkpoints, coded by non-mutated genes, are important non-oncogene addicts.
Master regulators	Protein participating in a modular regulatory structure, i.e., tumor checkpoint controlling the transcriptional state of a tumor cell. Master regulator proteins implement tightly autoregulated tumor checkpoint modules.

Pivotal pre-clinical experiments on tumor reprogramming highlight the potential therapeutic design space available for reprogramming tumors. Dysregulated homeostatic mechanisms, transcriptional programs and down-regulated tumor suppressor genes are important constituents of the tumor phenotype and available as keys to decisively change tumor phenotypes. Tumor cells in novel cellular environments, e.g., in embryonal stroma, act in phenotypically different ways in comparison to their original milieu. Suitable reconditioning of microenvironments may reprogram phenotypically plastic metastatic tumor cells toward a more benign phenotype (14). The extent of down-regulated tumor suppressor genes in tumor and stromal cells following oncogenic events, decisively determine the tumor phenotype (22, 37). Adult skin epithelium when exposed to different mutational and non-mutational insults develops dynamic cellular behavior for returning to a homeostatic state (16, 35).

Experimental models for reprogramming cancer cells to a more benign phenotype may not entirely mimic human disease in a clinical setting, but they may give us a line to a potentially extensive biological design space for therapeutically reprogramming tumor tissue. This may explain how therapies aiming at communicatively reprogramming tumor diseases, may exert a clinically relevant effect, even inducing continuous complete remission, by exploiting this design space, thereby transforming what is usually considered as non-curative care into a therapeutically efficient tool (9).

From pre-clinical observations, we can discern that tumor cells may secrete morphogens maintaining, for example, the pluripotency of embryonic stem cells (14), and that the cellular environment may reconstitute tissue homeostasis (16). Clinical results with therapeutic reprogramming reveal that **“master modifiers”** are available for functionally “normalizing,” or at least attenuating aggressive tumor phenotypes, in a clinically relevant way by interfering with communications-guided homeostatic processes in tumor tissue (8, 16).

### Communication Unifies All Systems Levels

Communication rules mediating environmentally conditioned communicative behavior are an important driving force for determining tumor phenotype (Figure 1). Combined with the specific interplay of either single or multiple genetic and epigenetic aberrations, they constitute unique disease-related hallmarks of cancer. Therefore, genetically different acute myelocytic leukemias constitute a unique phenotype, rapidly displaced normal hematopoiesis in the bone marrow. Accordingly, unique pro-anakoinotic therapy approaches may differentiate blasts from genetically different acute myelocytic leukemias into granulocyte-like cells (32, 34, 38–42).

A prominent tumor actor, mutated BRAF, highlights context-dependent communicative behavior: In different tumor types, for instance colon cancer and melanoma, BRAF has differential clinical activities. Treatment with BRAF inhibitors alone is only successful in metastatic melanomas (43, 44). Due to the communicative background of biological systems, gene mutations may not be consistently associated with important phenotypic disease characteristics. The communicatively altered state of a system, i.e., availability on demand at distinct time points, and current functional impact at a distinct systems stage, of potentially tumor-promoting pathways, make identification of new classic targeted treatments more difficult (Figure 1).

Important biological mechanisms may remodel the abundance and activity of modulatory protein hubs: **Super-enhancers** alter both the abundance and the functions and structures at the level of protein production, and scaffold proteins act at the level of protein localization addressing their activity (45–47). Cytokines, such as interleukin-6 or TGFβ, can switch their function during tumor progression from a growth inhibitor to a growth stimulator (45, 48). Additionally, heterotrimeric G proteins influence signal integration for regulating, for example, inflammation-related transcription factors (49).

Signaling pathways are modular, thereby facilitating therapeutic access. Modular binding domains of proteins are key points for altering signal transduction pathways in cells (50). Molecular domains or motifs characterize the link to input and output of signaling pathways; however, the catalytic activity of a signaling protein is functionally distinct from its domains or motifs underlining the high degree of modularity, and therefore adaptability (51).

## Anakoinosis: A Systems Biological Therapy Approach

Adjacent stromal cells cannot directly sense the oncogenic events in a neighboring tumor cell, but altered homeostatic mechanisms are recognized (16). Nevertheless, stromal cells may contribute to elimination or silencing of tumor cells. Thus, induction of anakoinosis, communicative reprogramming in diseased tissue for therapeutic purposes, is also a process that heterologous cell systems deploy under physiological conditions.

### Anakoinosis Induces Changes in the Flux of Information

Clinical trials of the combined administration of tumor phenotype targeting drugs characterize current anakoinosis research as a top-down approach (10, 11, 52). The top-down approach presupposes that the tumor phenotype provides non-oncogene addicted **master regulators** which are accessible for regulatory active drug combinations, for inducing tumor response (53).

Anakoinosis-inducing trials show that regulatory active drugs may mediate tumor response, that the response patterns indicate a regulatory communication-promoting activity profile. At the same time available biological and clinical response data indicate the administered drugs' activity profile can define them as **master modifiers of tumor tissue** (9). In addition to this clinical data, there is increasing biological data indicating master modifiers' mechanisms of action (22, 32, 41, 42, 54–56).

“Networked multicellular pharmacodynamics” describes anakoinosis pharmaco-dynamically as a model of how dysregulated transcriptional networks and tumor-associated signaling pathways may be, in concert, communicatively reorganized to attenuate tumor growth or to pave the way for continuous complete remission (57).

### Targeting Tumor Systems With Master Modifiers

**Figure 2:**
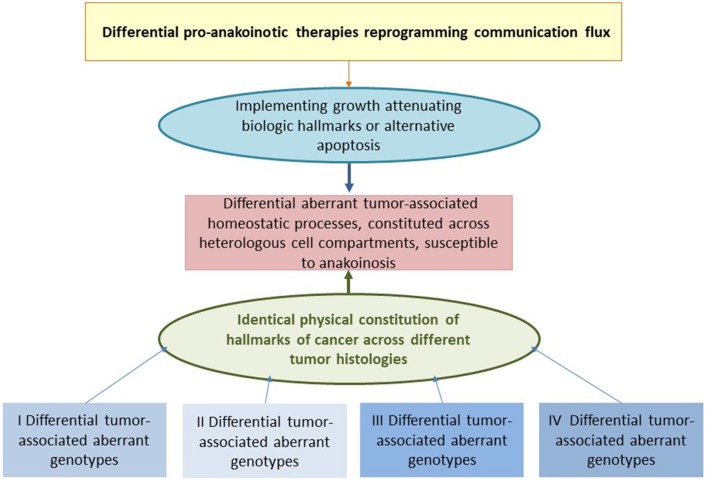
Reprogramming hallmarks of cancer via dysregulated homeostatic pathways and non-oncogene addictions. Tumor cells with diverse clusters of cancer signature genes generate in close interaction with adjacent stroma cells hallmarks of cancer via distinct physical constitutions of these hallmarks. Communication-technically described, the physical constitutions of hallmarks operate modules comprising master regulator proteins for maintaining combined with stroma cells the tumor cell state. Pro-anakoinotic therapies demonstrate that targeting patterns of non-oncogene addicted targets with combinations of master modifiers, may specifically change the communicative context, namely validity and denotation of systems participators, and finally, induce tumor response. Importantly, in contrast to multifold genetic clusters constituting unique hallmarks, for example rapidly displacing growth, these clusters might support only a restricted amount of constitutions for characteristic cancer hallmarks.

Therapeutic modulation of biological communications processes aims at prioritizing alternative pathways for apoptosis induction, normalizing activity of dysregulated homeostatic pathways, at up-regulating non-mutated tumor suppressor genes, attenuation of stroma-mediated support for tumor growth, and at modulating cancer checkpoints (Figure 2). The diversified activity profiles of **master modifier combinations** in tumor tissue, differentiation induction, transdifferentiation, control of tumor-associated, growth-promoting inflammation, immunologic control etc., indicate the broad therapeutic impact of anakoinosis (Figure 1). For example, Tables 2, 3 list the diversity of master modifiers, which may act up-stream and down-stream in signaling pathways of cancer and stroma cells. These drugs or drug combinations have shown the capacity for resetting intrinsic tumor communication processes, thereby facilitating the evolution of tumors into a more controlled phenotype (9).

**Table 2:** Specification of anakoinosis inducing therapies.

**Combinations with master modifiers**	**Model**	**Biologic effect**	**References**
Master modifiers	• Clinical trial, maintenance therapy	• Induction of biologic memory: Prolonging progression-free survival 2 (for lenalidomide maintenance)	(58)
Combined transcriptional modulators	• Clinical trials • Pre-clinical models	• Continuous complete remission possible	(10, 11)
Metronomic chemotherapy plus transcriptional modulator(s)	• Clinical trials • Pre-clinical models	• Continuous complete remission possible • Induction of biologic memory: Stable disease despite discontinuation of therapy	(8–11)
Epigenetically active drugs plus transcriptional modulators	• Clinical trials • Pre-clinical models	• Continuous complete remission possible • Regain of cellular functions	(10, 11, 32)
Targeted therapy (e.g., mTor, MEK inhibitor) plus transcriptional modulators	• Clinical trials • Pre-clinical models	• Differentiation induction • Complete remission	(10, 33, 59)
Pulsed chemotherapy plus transcriptional modulators	• Clinical trials • Pre-clinical models	• Enhancing efficacy of chemo-immune-therapy	(60)
Pulsed chemotherapy plus angiotensin receptor blocker	• Clinical trials • Pre-clinical models	• Reprogramming cancer-associated fibroblasts	(61, 62)
Immune checkpoint inhibitors (plustranscriptional modulation)	• Clinical trials • Pre-clinical models	• Bezafibrate increases or maintains the number of functional CTLs, leading to enhanced antitumor immunity during PD-1 blockade. • Metabolic reprogramming with PPARalpha agonists of CD8+ T cells increase energy production and improves treatment outcome upon PD-1 blockade	(63, 64)

**Table 3:** Glitazones plus targeted therapy for re-establishing tumor growth control.

**Targeted therapy**	**Model/tumor type**	**Biologic/clinical effect**	**References**
Rosiglitazone, MEK inhibitor	Mouse model; metastatic breast cancer	**Trans-differentiation:** Cancer cell plasticity can be exploited therapeutically by forcing **trans-differentiation** of epithelial-mesenchymal transition (EMT)-derived breast cancer cells into post-mitotic and functional adipocytes	(33)
Pioglitazone, mTor inhibitor	**Clinical trial** (plus metronomic chemotherapy, COX-2 inhibitor); Melanoma	**Prolonged progression-free survival:** Biomodulatory metronomic therapy in stage IV melanoma is well-tolerated and may induce prolonged progression-free survival, a phase I trial	(59)
Pioglitazone, all-trans retinoic acid	**Clinical trial** (plus azacitidin); *Ex vivo* experiments	**Differentiation** into functionally active granulocyte- similar cells in acute myelocytic leukemia	(32, 41)
Glitazones, EGFR TKIs	Cell culture; NSCLC	**Re-establish sensitivity to EGFR TKIs: via** PPARγ agonist-induced autophagic cell death, and up-regulated PTEN	(65)
Glitazones, vemurafenib	Cell culture, circumvention of vemurafenib resistance; Melanoma	**Sensitizing:** Rosiglitazone increases klotho and decreases Wnt5A in tumor cells, reducing the burden of both BRAF inhibitor-sensitive and BRAF inhibitor-resistant tumors	(66)
Glitazones, imatinib	**Clinical trials**; *in vitro* experiments; Chronic myelocytic leukemia (CML)	**Molecular complete remission (CR):** Glitazones decrease expression of STAT5 and its downstream targets HIF2α and CITED2, which are key guardians of the quiescence and stemness of CML leukemia stem cells (LSCs)	(54)
Glitazones, Selenium	Cell culture; CML	**Leukemia stem cell quiescence:** Activation of PPARγ by endogenous prostaglandin J_2_ mediates the antileukemic effect of selenium in murine leukemia. Selenium-dependent activation of PPARγ, mediated by endogenous CyPGs decreased Stat5 expression leading to the downregulation of Cited2, a master regulator of LSC quiescence	(67)

*Expedient therapeutic approaches seem to be combinations of transcriptional modulators, such as pioglitazone, with inhibitors of non-oncogene addicted targets, e.g., mTor, MEK inhibitors etc. Additionally, pioglitazone may up-regulate, for example the tumor suppressor PTEN*.

Network-guided therapy is the backbone for the combinatorial use of master modifiers. Anakoinosis-inducing therapies reorganize mechanisms underlying the tumor's robustness (68). In cancer tissue, communicative networks and their physical constituents are strongly geared to stabilizing tumor growth. Even the removal of major hubs does not collapse the tumor system and tumors may maintain the capacity for self-organization (69, 70).

Biomodulatory therapies mobilize important aspects of the tumor's available “background knowledge” or “system state” as a process that may allow the establishment of hallmarks associated with attenuation of tumor growth up to remission induction, suggesting possible “signatures” (Table 1). The communication-mobilizing capacity of anakoinosis-inducing schedules could be the reason why those schedules are still active in resistant, advanced disease.

The orchestrated regulatory activity profile of master modifiers is based on developmental programs active during tumor ontogenesis: dysregulated transcription programs, networks of pathways and interlaced communication routes among cancer cells, adjacent stroma cells, tumor bearing organ and organism. Thus, across different histologic tumor types distinct hubs arise, tumor checkpoints or constitutions of the hallmarks of cancer, and alternative communication routes. These systems targets develop secondarily to the oncogenic events due to distorted communication processes and are precious “actionable” targets for tumor control. They lend themselves as targets for master modifiers, facilitating the reset of tumor tissues' homeostasis and the reorganization of communicative contexts in a therapeutically relevant way (9–11, 32, 41, 71–79).

An important characteristic of anakoinosis has been that master modifiers also comprise agonistically active drugs, often with only partial or no single-agent activity, but presenting a concerted activity profile in combination (9–11).These drugs must be administered in regulatory active concentrations and not at maximum tolerated doses (80). **Agonistically active drugs**, such as transcriptional modulators, contribute to the orchestrated restoration of apoptosis or differentiation competence (32, 41) at **low, regulatory active dose levels** and in a clinically relevant way (9, 81). Communication-guiding therapy aims to facilitate the reversing of tumor conditioning, also attenuation of aggressive tumor growth, as well as resolving tumor-associated conditions at distant organ sites, e.g., resolving cachexia (18, 59).

As indicated in Table 3, the peroxisome proliferator-activated receptor alpha/gamma agonist pioglitazone, an agonistic master modifier, establishes in combination with classic targeted therapies completely novel therapeutic effects in pre-clinical models or clinical applications.

### From Off Target Effects and Side Effects to Drug Repurposing

**The off-target activity** of a drug may be both a direct and a communicatively mediated indirect biologic activity that is different from the intended activity and obviously initiated by alternative drug targets. Pivotal examples for an off-target effect represents, individual pharmacokinetics and –dynamics etc. The off-target effects are often only obvious in an altered therapeutic context, as shown for pioglitazone and its contribution to cancer control (11).

Physicians can take advantage of drugs' off-target activity for establishing pro-anakoinotic therapy regimens, as shown for the master modifier examples. The therapeutic rededication of a drug is called **drug repurposing** (82). Thus, drugs can be administered in completely novel combinations while considering their potential for communicative reprogramming tumor systems biology, together with their off-target effects (83). Moreover, completely novel “team players” may be added, even players with no monoactivity in malignant disease, but which contribute to the concerted regulatory activity of a drug cocktail (Table 3).

A possible “off-target” effect seems to be the induction of tumor cell death and continuous complete remission following anakoinosis inducing therapies. For example, while controlling refractory AML with pioglitazone, all-trans retinoic acid and azacitidine, differentiation induction, and gain of function of blast-derived granulocytes may not be the only mechanisms explaining continuous complete remission, even molecular remission in single patients (41, 42).

### Frequent Metronomic Dosing of Pro-Anakoinotic Therapies

Anakoinosis inducing therapies frequently use **metronomic scheduling of drugs** (84, 85). However, metronomic dosing is not a prerequisite (41, 60, 86). Metronomic scheduling of drugs creates a more tolerable regimen and can ensure a continuous level of regulatory activity.

The main point of anakoinosis is the appropriate therapeutic use of the tumors' design spaces according to universally available communication rules as presented in Figure 1. Due to still missing pre-therapeutic diagnostic tools for evaluating an “evolution-adjusted tumor pathophysiology” (87), protocol designs are mostly developed empirically. Systematic structuring of trial designs, however, are leading to a deeper understanding of how anakoinosis works (9, 32, 33).

Therefore, the major challenge is to account for the complexity of communicatively linked compartments in a tumor with respect to pro-anakoinotic therapeutic interventions. The intention to treat pro-anakoinotically must be principally separated from reductionist ones focused on combinations of targeted therapies. In contrast to biomodulatory therapies, these approaches are concerned with “shutting off” and “knocking down,” and therefore, characteristically struggle with mechanisms promoting the tumor's robustness and resistance (69). Pro-anakoinotic therapies simultaneously target tumor and stroma cells and therefore, may serve as an essential strategy for overcoming therapy resistance supported by the tissue microenvironment (36).

### Targeting Physical Constitutions of Hallmarks of Cancer

Communication tools enable the therapeutic realignment of endogenous tumor-associated evolutionary processes. The hallmarks of cancer constitute tumor-associated evolutionary processes via diverse, and often unspecified, physical constitutions. Interindividual phenotypically heterogeneous melanoma cells with the specific capability for metastasizing in the brain, as indicated by proteome analysis, highlight, for example, the need for diagnostically specifying diversified physical constitutions of a distinct tumor-associated hallmark and for enabling specific targeting of the metastatic melanoma's organ-tropism (2).

Clinical observations of anakoinosis-inducing therapies reveal that tumor tissues provide an extensive design space, including the interaction of tumor and tumor-bearing organ and organism (9). The way in which tumors respond with clinically meaningful changes in tumor phenotype following exposure to identical combinations of master modifiers may be similar across different histologic tumor types. Thus, unique communication tools are available for maintaining identical hallmarks of cancer within different tumor histologies. These clinically derived observation give rise to a novel categorization of “physical constitutions” of hallmarks of cancer across different tumor histologies in an “evolution-adjusted” tumor pathophysiology (87, 88) (**Figure 4**).

Tumors may establish, unique physical constitutions for hallmarks of cancer for example, immune responses at different metastatic sites, although genetic or molecular-genetic heterogeneity would suggest heterogeneous physical constitutions in case of microsatellite instable tumors (89, 90). Thus, anakoinosis inducing therapies may principally overcome that major obstacle of classic targeted therapies, namely genetic tumor heterogeneity (90).

### Biological Memory: Changes in Tissue Phenotype Following Tumor Therapy

Each tumor therapy leaves biological “traces” in the tumor tissue. The “traces” comprise highly divergent biological phenomena, such as tumor resolution or defect healing, stimulation of tumor growth via DNA-damage and consequent apoptotic cells, senescence, development of drug resistance etc. Clinical traces can include long-term tumor control, as indicated by PFS, OS or progressive disease, or in the long run by secondary malignancy.

Biological “traces” and side effects may be part of the reason that sequential therapies must follow distinct sequencing of drugs, and that efficacy in first-line cannot be rescued by administering an approved first-line therapy as second-line, for example in case of checkpoint inhibitor in NSCLC (91).

At the end of the therapeutic cascade of sequential anti-tumor therapies, we consistently find high-risk disease, which is characterized by an evolutionary trajectory that has imparted multiple “traces.” Typically, diagnostics are insufficient to work up such end-stage diseases for adapted therapy. At end-stage disease, evaluation of singular highlighted molecular-genetic mechanisms, as initiated in molecular tumor boards, without considering the communicative context, is rarely able to define decisive therapeutic targets in end-stage tumor disease or the risk profile of a tumor disease, which is then characterized by apoptosis resistance, acquired drug resistance etc. (92). Diagnostic procedures commonly do not focus on the impact of cellular functional changes in tumor “ecosystems,” which finally facilitate aggressive tumor growth.

Specific sequential therapies can contribute to improved long-term survival in metastatic solid tumors and hematologic malignancies (93). Changes in the tumor following administration of sequential therapy rarely prompts for diagnostics to help specify the next-step therapy. Yet understanding the tumor's clonal evolution and the associated tumor phenotypes would seem to be pivotal for a rational design of sequential therapy (4). Frequently clinical needs, particularly the need to control comorbidities, are dictating *n*-line therapies. However, in end-stage disease modification of the basic setting of the tumor-stroma-organ interaction with master modifiers seems to be, as has been shown, particularly important therapeutically.

Biomodulatory effects of pro-anakoinotic therapies facilitate induction of **biologic memory** (9, 10, 94). Prolonged therapy-free intervals without significant progression following discontinuation of study therapy or prolonged survival are indicators of such biological memory. In contrast to poor PFS in cases of therapy with immune checkpoint inhibitors, OS may be noticeably prolonged (9, 56).

Biological memory also summarizes all “traces” left by sequential therapies, finally aggregating in evolutionary processes which are characteristic of resistant end-stage disease (95–97). And vice versa, biomodulatory therapies may keep in check metastatic disease (9, 94).

Tumor therapies may also give rise to secondary malignancies with specific molecular-genetic characteristics and may evolve tumor systems by leading to even more genetic heterogeneity compared to the initial diagnosis. Clinically, a disease at relapse or progression may be like that of initial diagnosis but it may also present with a novel clinical disease pattern.

### Anakoinosis: Novel Outcome Parameters and Prognostic Parameters

As indicated in Table 4, pro-anakoinotic therapies represent a broad spectrum of approaches combining agonistic or antagonistic master modifiers reaching non-oncogene addicted targets in tumor, stroma cells and tumor harboring organs (Tables 2, 3) (105–109). Due to the possibility of targeting quite different communication fluxes in tumor systems with well-tuned combinations of master modifiers, pro-anakoinotic therapies may induce multifold biological and clinical read-outs, as shown in Table 4. These may extend beyond classical response parameters usually monitored following therapies directed toward oncogene-addicted targets.

**Table 4:** Diversification of non-curative care by re-establishing growth attenuating biologic hallmarks via pro-anakoinotic processes.

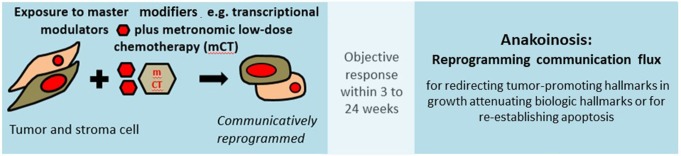	
**Re-establishing growth attenuating biologic hallmarks or apoptosis**	
**Combinations of master modifiers: resetting tumor systems**	**References**
**Biologic outcome: Changes in tumor biology** • Simultaneous modeling of heterologous cell compartments and pathways • Tumor stem cell quiescence: Targeting the tumor cell niche • Simultaneous inflammation control, anti-angiogenesis, immunologic tumor control, modeling of tumor metabolism • Targeting dysregulated homeostatic pathways • Targeting tumor system's robustness • Induction of differentiation with regain of function, transdifferentiation, biologic memory • Therapy effects beyond therapy discontinuation: Induction of biologic memory • Attenuation of metastatic spread or outgrowth **Clinical outcome: Interactions of cellular compartments, tumor-organ and -organism interactions** • “Off-target” effect: Tumor cell death followed by continuous complete remission (alternative pro-apoptotic pathways) • Resolution of cachexia while stabalizing metastatic tumor disease • Favorable effects on efficacy of consecutive therapies (progression-free survival 2) • Pro-anakoinotic therapies replace temporary complete remission or molecular complete remission by long-term disease stabilization at minimized toxicity (replicative arrest or tumor dormancy) • Inhibition of further metastatic spread following progression after pro-anakoinotic therapy • Tumor control via resetting interaction of tumor harboring organ and tumor	(8–11, 18, 33, 54, 58, 59, 94, 98–104)

*Master modifiers facilitate reprogramming of tumor tissue, for example via agonists of nuclear transcription factors, which exploit—from a communication technical view—the tumors' “background knowledge”*.

An important aspect of novel therapy methodologies, such as anakoinosis induction, is that common prognostic markers associated with established systemic tumor therapies may need to change.

For example, only the combination of two master modifiers, all-trans retinoic acid and arsenic trioxide, abrogate the negative prognostic impact of FLT3-ITD in promyelocytic leukemia, whereas all-trans retinoic acid alone may not impede the negative influence of FLT3-ITD (55, 110). More generally, anakoinosis-inducing therapies may work in resistant metastatic tumor disease, thus may overcome drug resistance and resistance due to the progressive genetic instability developed by following sequential tumor therapies.

Induction of complete remission in p53 mutated acute myelocytic leukemia (AML) with pioglitazone, all-trans retinoic acid and azacitidine demonstrates that undruggable targets may be by-passed by activating alternative druggable pathways (42, 71). In refractory AML and advanced multiple myeloma patients responses in the form of long-term disease stabilization can be achieved with metronomic scheduling of master modifiers, even in case of complex karyotypes (42, 111).

## Descriptive Considerations on the Role of Communicative Reprogramming in Tumorigenesis and Response to Treatment

The diversity of biological and clinical responses to master modifiers in tumor tissues allow us to descriptively delineate the communication rules as indicated in Figure 1. Prerequisite for the conclusive description of communication rules are the following findings from therapy of metastatic resistant tumor disease using master modifiers.

- Clinical responses can be achieved without therapeutically targeting *any* driver mutations in tumors.
- Master modifiers are not direct cytotoxic and do not necessarily induce apoptosis in producing a positive tumor response.
- Low drug concentrations are sufficient for establishing favorable clinical outcomes, revealing concerted biomodulatory activity.
- The combined use of regulatory active drugs in low concentrations is enough to induce tumor control. Due to the regulatory activity profile of master modifiers maximum tolerable doses can be omitted (10).
- Different histological tumor types share communications-related characteristics due to cross-reactivity of combinations of master modifiers among different tumor histologies. As indicated in Table 2, different pro-anakoinotic approaches aiming at different biological outcomes in identical histologic tumor types may be available (8, 10, 11, 33, 112, 113) (Tables 2, 4, Figure 1).
- Combinations of master modifiers are more efficacious in tumor control than mono-therapies and may even induce continuous complete remissions in therapy-resistant metastatic tumors or hematologic neoplasia.
- Biological and clinical heterogeneity of systems responses characterize a broad diversity of non-curative important outcomes, including among others, inhibition of metastatic outgrowth at novel metastatic sites in case of tumor progression, long-lasting systems responses without therapy, differentiation induction and transdifferentiation.

### Remodeling Metastatic Tumors: The “System State” of Cells, Tissues, and Organs as Therapeutically Accessible Communication Tools

Cell compartments and tissue systems, organs and organisms have the capability to respond with their intrinsic communication tools to communications-driven challenges promoted by oncogenic events or pro-anakoinotic therapies. The endogenous or extrinsic challenges may activate a broad variety of response patterns, i.e., acute and chronic evolutionary processes as indicated by rapid and alternatively delayed response or response beyond discontinuation of therapy (**Figure 4**). Endogenous challenges are associated with either the establishment of hallmarks of cancer or control of cells with oncogenic load; extrinsic therapeutic challenges with the implementation of biological hallmarks that keep tumor growth under control, i.e., with repair mechanisms (*reconstitution ad integrum* or defect healing), tumor cell apoptosis or death of the organism.

The sum of possible tissue response patterns represents the respective systems' “background knowledge” or “system state.” This communicative “background” tool supports robustness, evolvability and repair within cellular systems following endogenous or therapeutic, e.g., pro-anakoinotic activation (Table 1).

In any case, communication in tumor systems is a multiscale modeling (Figure 1). Tumor cells do not exclusively guide tumor-associated communication processes by themselves. Phenotypic outcomes of tumor disease depend equally on communication networks constituted by both cells with potential oncogenic aberrations and by neighboring stroma cells and organs, even by distant cell systems and organs. Consequently, pivotal changes in tumor-associated stromal cells may have prognostic relevance (114).

The presence of oncogenic as well as pro-anakoinotic therapeutic events challenges the system state and provides the chance to study how it provides responses to endogenous or therapeutic stimuli. Thereby, a biological system discloses timely and locally unique communications-derived reactivity profiles.

The reason why a distinct system state exists may be figured out by the correlation of systems' structures, functions and hubs with reproducible systems-associated phenomena, i.e., diverse biological or tumor-associated hallmarks. Communication rules and techniques organize the interface between extrinsic and endogenous stimuli or perturbations and the available response patterns of cell compartments, tissue systems and organs: Therefore, outcomes are not necessarily predictable, and may depend to high degree on local and time-dependent cellular and communication-technical configurations (9). The individual tumor phenotype may deviate from canonical descriptions of a disease entity. The difference between theory and practice describes this apparent discrepancy (87).

### System State and Responses to Challenge

In a further step, we must explain how the system state of cells, tissues and organs relates to the response to challenge. This handling of risk or response to challenge is particularly underestimated therapeutically even though it is routinely activated following therapeutic intervention. It is after a therapeutic intervention that previously “silent” molecular processes or pathways embedded in the system state are activated—thus ensuring system robustness if the face of challenge. A particularly pertinent example is shown by the re-growth of tumor following therapeutic interventions and consecutive apoptosis induction.

Concerns have been recently raised by the unexpected findings that caspase-3 and caspase-7, the proteolytic enzymes responsible for orderly cell dismantling during apoptosis, also activate extracellular signals, being major players of cancer cell re-population after cytotoxic therapies (115). Caspase-3, *via* proteolytic activation of phospholipase A2, elicits prostaglandin E2-mediated pro-survival paracrine signals (116), which coordinately promote the proliferation of the cancer cells surviving the cytotoxic treatment. Dying cells thus elicit a response aiming at protecting the tumor microenvironment, projecting its regeneration: hence the term **“phoenix rising”** (117–119), phoenix being the mythological bird that re-grows from its own ashes. This apparently paradoxical effect is in fact an evolutionarily conserved response to tissue damage (120), allowing setting up effective strategies to homeostatically repopulate an injured tissue (121). Caspase-dependent apoptosis thus plays the basic and perhaps unexpected role of maintaining tissue homeostasis, coordinating cell death with proliferation, thus assuring restoring the correct organ size (122). Caspase-3 is the fundamental regulator of organ regeneration in lower animals (e.g., no regeneration in caspase-3^−/−^ planarias (123), and a major player in liver regeneration and skin wound healing in mammals (124). The tumor environment, which possesses aberrant, but highly organized homeostatic rules, behaves and reacts as any regular tissue that, after abundant cell loss, must take care of restoring proper size and functionality.

Caspase-dependent apoptosis, considered as inflammation-silent for decades, now looks rather as an anti-inflammatory and immuno-suppressive process (125). Corresponding studies suggest that the establishment of a tolerant, proliferative state after cytotoxic therapies may nullify the antitumor effects of the therapy by promoting tumor re-growth. In cancer patients, the degree of caspase-3 activity in tumor correlates with unfavorable prognosis (126), pointing to consider the reported experimental evidence as clinically relevant, and leading to establish **adjuvant pro-anakoinotic therapies** aimed at inhibiting caspase-3 (58, 127) or PGE2 (128) during radio- or chemotherapies to prevent tumor re-growth and reduce the risk of relapses.

During spontaneous tumor remission, the loss of tumor cells has been shown to occur through caspase-independent apoptosis (129): this is clinically relevant, because it suggests that less aggressive means of inducing apoptosis might be fruitfully adopted also in therapeutic approaches. The important message implied is that not all apoptotic types of death are equivalent (130) in terms of clinical efficacy. The number of cells killed is not the only relevant parameter; also, the consequences put in motion by the cell death process itself must be considered when programming a therapeutic treatment. Interestingly, oncogene-induced apoptosis, which is the natural way of pre-cancerous cell elimination, or that induced by reprogramming therapies, may occur without caspase activation (131–133).

### Holistic Communicative Context

The availability of master modifiers for guiding the tumor's phenotype (Table 2), demonstrate that communicative events, as described in Figure 1, tie the **holistic communicative activity** of a tumor, while simultaneously modulating heterologous cell types within it, thus altering the tumor's phenotype in a therapeutically significant manner. Structures, functions and hubs in a tumor system are matched at each time point, even if in a distorted manner, and therefore, contribute to the uncertainty about the functional status of each systems component within a tumor at a distinct time point and localization (9). Holistic biological systems are self-regulated open systems, displaying both autonomous and dependent properties within distinct subsystems (134).

Also, deterministic ideas about the development of aberrant genetic patterns in cancer should not hide the fact that intercellular communication is unbiased as to the result within the framework of a particular system state (135). Thus, genetic tumor evolution, as well as communicatively developing non-genetic and cell non-autonomous diversity, may influence susceptibility to systems-targeted, pro-anakoinotic therapy. For example, network-specific drug targets have been suggested to maximize undruggable targets, such as p53, via network-mediated cell death (136).

Objectifying the multiscale modeling of pathophysiological or therapeutically intended evolutionary processes could derive a “social” action theory still starting with oncogenic events, but now considering how they are embedded in complex communicatively developing contexts defining the tumors' therapeutically accessible phenotypes. Mathematical and biological considerations may equally contribute for depicting phenotypic complexity. Simultaneously assaying chromatin accessibility and the transcriptome within the same single cell will provide more accurate information about its' functional stage within an evolutionary process and will enable deciphering communication-derived heterogeneity of complex heterologous cell populations (137).

Addressing the question of which background communication processes initiate tumors first, for instance, to alter the validity and function of transcriptional processes, requires a clarification of the single steps of communication from an *intentional point of view* (communication theory) (87, 138). An analysis of the prerequisites for communicative action seems to be necessary to exploit the diversity of the background of the tumor's “living world,” which cross-links and stabilizes larger cell communities.

### Evolutionary Processes in Tumors

Tumors develop under the selective pressures from a broad range of homeostatic dysregulations on the one hand and a considerable robust response based on acquired oncogenic drivers. These pressures are also therapeutically important, for example by an increase in genetic instability and heterogeneity during tumor progression (139). Nevertheless, the tumor disease may re-appear with identical symptoms compared to those at primary diagnosis, although the genetic background in tumor cells has changed.

The development of evolutionary processes in tumors can be summarized as the sum of extrinsically, i.e., therapeutically, and intrinsically inducible evolutionary processes within the respective tumor environment (tumor stroma, hosting organ, distant organ sites) (Figure 1). The adjacent tumor environment does not contribute as simple bystander but interacts with heterogeneous cell compartments and initiates novel structures, functions and hubs. The degree to which this happens varies by type of cancer. Tumor systems are on the move between tumors with about 5% tumor cells (Hodgkin disease) and those with 90% tumor cells, for example acute leukemias in the bone marrow (140, 141).

Evolutionary processes in tumors may not be due solely to novel mutations but may be also be due to altered epigenetically regulated gene expression profiles and are dependent on the available neighboring stromal cell compositions contributing to the identity of respective cell types (87).

### Tumor Checkpoints as Communication Tools and Pharmacological Targeting

Anakoinosis-inducing therapies may induce response rates of more than 60% in histologically quite different tumor types and refractory metastatic disease (9, 10, 38). That there is a group of non-responders to master modifiers indicates the specificity of a distinct systems-targeted, pro-anakoinotic therapy approach. Different genetic clusters constitute different tumor phenotypes based on oncogene-independent dysregulated proteins and homeostatic pathways (Figure 2) and presumably support the growth of non-responsive tumors. Indeed, adapting the composition of master modifiers in non-responders may invoke a response, for example in renal clear cell carcinoma or Langerhans cell histiocytosis (9).

Figure 2 summarizes the working hypothesis about systems-related tumor targets and the activity profile of master modifiers. Data on anakoinosis-inducing therapies suggest that master modifiers may target superordinate regulators of communicatively linked tumor checkpoints. In other words, auto-regulated modules comprising master regulator proteins, e.g., STAT3, STAT5, RUNX, NF-κB etc. that maintain tumor cell state together with master regulators in the adjacent tumor microenvironment (142–144). Tumor checkpoints are auto-regulated modules comprising master regulator proteins maintaining tumor cell state. Califano et al. summarized a similar working hypothesis arising from a bottom-up approach, supported by molecular-biological data (145).

Different genetic clusters within a histologically homogeneous group of tumors may support, via different physical constitutions of the hallmarks of cancer, various patterns of non-oncogene addicted systems-targets (146, 147). These are accessible for specific combinations of master modifiers, which facilitate converting the hallmarks of cancer into biological hallmarks associated with the attenuation of tumor growth, or finally, apoptosis induction (Figure 2). Additionally, distinct physical constitutions may be common across quite different tumor histologies. The therapeutic efficacy of master modifiers demonstrates the regulatory and signaling interactions of master modifiers with master regulators and homeostatic pathways among both tumor and stromal cells.

Classifying the dysregulated regulatory architecture of tumors may provide a conceptual framework for functionally elucidating, at the level of tumor phenotypes, the complexity and heterogeneity of genetic clusters among histologically defined cancers.

### Correcting Aberrant Homeostasis of Cancer Tissue

**Figure 3:**
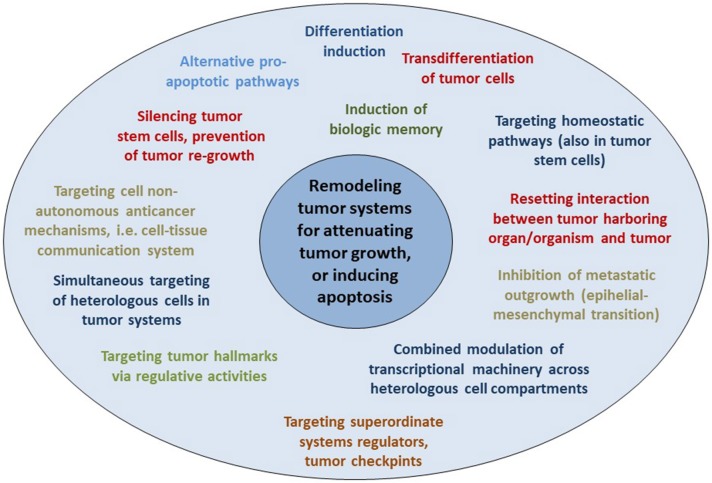
Resetting information flux in tumor disease. Multifold possibilities for resetting information flux in tumors with master modifiers for establishing long-term tumor control in metastatic resistant disease.

Apoptosis induction via tissue-promoted pathways (as opposed to direct drug-promoted cytotoxicity) may be a mechanism of action of pro-anakoinotic therapies. However, diverse therapy outcomes (Figures 2, 3) reveal that long-term tumor control is frequently linked to novel mechanisms of action. Transdifferentiation, differentiation induction, reprogramming of the whole hematopoietic compartment in case of acute myelocytic leukemia, interaction of tumor, and the involved organ (in case of hepatocellular carcinoma), replicative arrest associated with long-term stable disease are appropriate mechanisms for the control of metastatic, even resistant tumor disease (10). Moreover, pro-anakoinotic therapies might switch the method of inducing different cell death modalities, probably by promoting **physiologically-induced apoptosis** by perturbations of the adjacent tumor microenvironment (148). Evidently, alternative pro-apoptotic pathways might also play a role in pro-anakoinotic strategies, otherwise long-term continuous complete remission could not be achieved in resistant metastatic tumors (71).

Peroxisome proliferator-activated receptor γ (PPARγ) agonists, master modifiers of lipid biology are bridging lipid and carbohydrate metabolism with the regulation of transcription factors involved in tumor growth (11, 76, 102, 149–152). While introducing pioglitazone as pro-anakoinotic therapy component in resistant metastatic tumors, clinical results indicate that diversified mechanisms for tumor control may be established by glitazones (Table 3). The clinical observation that pioglitazone in combination with other master modifiers may overcome therapy resistance could suggest that glitazones may also induce alternative cell death pathways (153). Thus, targeting driver mutations and acquired resistance plays a minor part when choosing pro-anakoinotic therapy models.

In chronic myelocytic leukemia, pre-clinical and clinical data clearly show that PPARγ agonists may target the leukemia stem cell population (54, 154). Additionally, PPARγ agonists as master modifiers participate in the modulation of epithelial-mesenchymal transition as indicated in an animal model (33, 103), target multiple homeostatic pathways, particular in tumor stem cells (155) and inhibit the development of senescence (156, 157).

Metronomic low-dose chemotherapies introduce the possibility for inducing alternative pro-apoptotic pathways, thus facilitating circumvention of apoptosis resistance (158).

### Mathematical Model on Anakoinosis

Currently, pro-anakoinotic therapy strategies cannot be mathematically modeled as top-down approaches by using Bayesian networks, co-expression networks or module-based approaches. The fundamental biological systems parameters explaining complex tumor response patterns cannot be sufficiently provided, for example the multidimensional communicative interactions of cancer cells, adjacent stromal cells and cells of the tumor harboring organ (7, 159, 160).

Thus, a mathematical description of anakoinosis at the current level of knowledge regarding biological mechanisms of action seems to be problematic. However, anakoinosis may be modeled computationally using conceptual tools such as systems state, communications protocols between components representing diverse cell types and between cells and tissues (118, 119). Such a communications-derived model indicates that the cell-tissue communication system may act as an intrinsic non-cell autonomous anticancer mechanism. Results from this model are in line with clinical observations from pro-anakoinotic therapies and must be now related to biologic processes in tumor tissue within a translational approach (Figure 3) (10, 118). Such communications-led models are an important tool for elucidating the complex inter-play between different cell types in and outside the tumor, particularly as the relationship between cell populations (stromal, immune, cancer etc.), are yet to be comprehensively formulated.

## Perspectives

### Anakoinosis: Diversifying Non-curative Care

Response rate and progression-free survival (PFS) are, in the short term, important parameters for estimating the efficacy of tumor therapies. However, biomodulatory therapies are increasingly available, including master modifiers, affecting particularly progression-free survival 2 (PFS2). That means that pro-anakoinotic therapies administered as pre-treatments, influence the outcome of subsequent therapies, contrasting tumor progression (58, 142, 161, 162). Pro-anakoinotic therapies lead to long-lasting responses through long-lasting changes in tumor tissue and by modifications of the tumors' communications-mediated system state (Figures 2, 3). Interestingly, biomodulatory drugs, such as lenalidomide, checkpoint inhibitors and other pro-anakoinotic schedules give rise to these long-lasting growth-attenuating effects.

As shown in clinical trials, therapeutically induced communicative-reprogrammed processes may persist after discontinuation of therapy (biologic memory): In castration resistant prostate cancer, a persistence of response was observed following anakoinosis-inducing therapy after discontinuation of therapy, for >1 year, although tumors had initially had a rapid PSA doubling times of <3 months (9). Durable responses after discontinuation of pro-anakoinotic therapy were also found in relapsed/refractory multiple myeloma (94). The add-on of anakoinosis-inducing therapy to classic targeted therapy may even eradicate chronic myelocytic leukemia (54) (Table 3).

These observations indicate that anakoinosis-inducing therapies improve clinical read-outs by contributing to novel, and yet therapeutically underestimated, biological behaviors in cancer, such as biologic memory (Figure 3).

Long-lasting therapeutic effects that extend beyond therapy discontinuation indicate that it is possible to further improve non-curative care with master modifiers (Figure 3). Long-term remissions may be induced in histologically different tumor diseases in response to checkpoint inhibitors or other master modifiers of tumor tissue. For example, continuous complete remission following anakoinosis-inducing treatments has been observed in angiosarcoma, renal clear cell carcinoma, acute myelocytic leukemia, and Langerhans cell histiocytosis (9, 10).

Additionally, pro-anakoinotic therapy seems to be an option in the adjuvant therapy setting for either preventing the re-growth of disseminated tumor cells or for inhibiting the growth-promoting activities of apoptotic cells (118, 163).

### Chemoprevention

Induction of anakoinosis may play an important role in chemoprevention. Master modifiers, such as glitazones, could be important agents for preventing carcinogenesis or particularly, as recently shown, tumor progression. The reset of tumor systems behavior and the redirection of developing hallmarks of cancer could generally play a decisive role in the process of chemoprevention (10). The capacity of master modifiers for inducing biologic memory could be an additional important therapeutic aspect. Biomodulatory therapy approach with lenalidomide in combination with pioglitazone, dexamethasone, and metronomic low-dose chemotherapy with treosulfan in patients with relapsed/refractory multiple myeloma > second-line (9, 94).

The fact that pro-anakoinotic therapies may provide access to complex communication systems offers the chance to learn from clinical trials of the chemopreventive activity of anti-diabetic drugs, like pioglitazone or metformin (155, 164–166). For example, pioglitazone may prevent carcinogenesis in case of exposure of mice to cigarette smoke (167).

### Germline Mutations and the Primed for Cancer Hypothesis

The current experience is that germline mutations irreversibly prime patients for cancer. Anakoinosis principally provides the theoretical background for a chemoprevention model, as well as derived from interventional or maintenance data, the clinical background for conferring the option to reducing the risk for developing cancer in patients with germline mutations. Thus, potential drugs reducing the risk of cancer, for example in case of TP53 or BRCA germline mutations, number among chemopreventive agents (168). There is an urgent need for studying the clinical benefit of master modifiers in patients with germline mutations.

Master modifiers may intervene in communication-driven homeostatic processes between epithelial/mesenchymal/hematologic cells and adjacent stroma cells for preventing the formation of possible tumor initiating niches (169). Peroxisome proliferator-activated receptor gamma and retinoids have already shown in animal models chemopreventive effects (80, 170, 171). It is suggested, that pharmacological interventions are capable of altering pre-cancerous niches, thus potentially reducing the cancer risk in individuals with germline mutations (168).

### Reverse Anakoinosis: Cancer Induction via Communicative Reprogramming

A current well-reasoned hypothesis implies that synergism of non-carcinogens may give rise to tumor development (172, 173). Based on successful pro-anakoinotic therapy approaches, it seems feasible the reverse, i.e., that the concerted activity of non-carcinogens with biomodulatory activity profile may modulate sequences of key events thereby promoting the classic hallmarks of cancer in target tissues. Interestingly, non-carcinogenic chemicals, ubiquitously present in the environment, have been already identified as potential promotors of hallmarks of cancer (172, 173).

**Figure 4:**
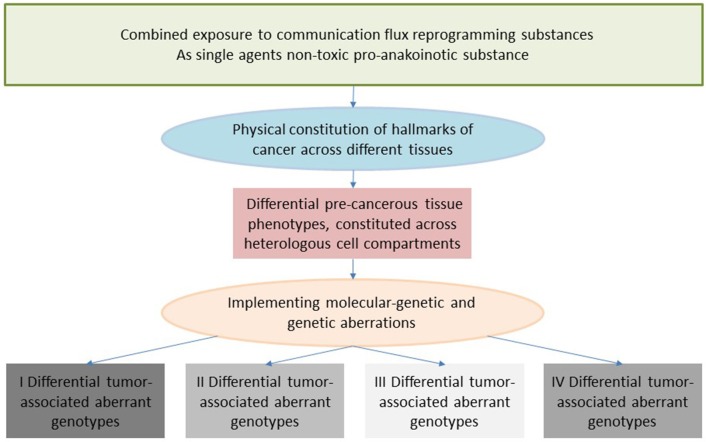
Reverse anakoinosis, i.e., induction of oncogenic events via concerted activity of as single substances non-oncogenic agents.

The concerted activity of non-carcinogens therefore could end up in a carcinogenic activity in target tissues besides the development of “off-target” effects. Cigarette smoke contains thousands of chemicals, including many known carcinogens (nitrosamine, arylamines) (174) and suggested non-carcinogens (nicotine) and induces, in experimental models, toxicological changes. The contribution of each single component on cancer induction is difficult to pin down (175). In smokers, non-small cell lung cancer may be induced besides “off”-target effects, such as chronic obstructive pulmonary disease (COPD), vascular disease etc. (Figure 4) (176, 177).

Observations of anakoinosis inducing therapies support the hypothesis of reverse anakoinosis, which would have far-reaching implications for environmental toxicology (178). In addition, induction of treatment-associated tumors by preceding systemic therapies might be explained by reverse anakoinosis, as for example in Hodgkin's disease (179). Treatment-associated tumors are a frequent problem of systemic chemo/radiotherapy (180).

Reverse anakoinosis would not call into question genetic or molecular-genetic events as primary events of final tumor development. The communication aspect would place special emphasis on cell non-autonomous communication-driven changes preceding tumor development via primarily phenotypical changes enforcing secondary genetic alterations (181, 182).

## Discussion

For depicting the communications processes in tumors, or between tumors and hosting organ or organism, we need basic communication, phenotypic and genetic descriptions, for putting respective data in a systems context and for creating mathematically derived models unifying bottom-up and top-down approaches.

**Figure 5:**
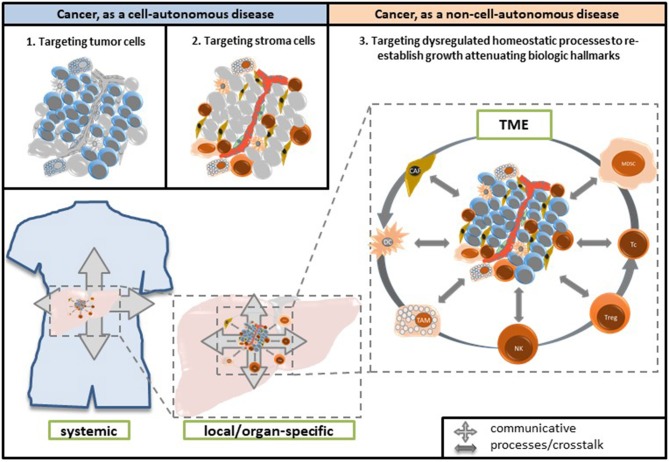
For therapeutic considerations, cancer is currently considered as a cell-autonomous disease. Successful administration of anakoinosis inducing therapies indicates that neoplastic cells are also non-cell autonomous: Targeting homeostatic pathways and “normalizing” their dysregulation in neoplastic tissue may be sufficient for inducing long-term response, even continuous complete remission. TME, tumor microenvironment; CAF, cancer-associated fibroblasts; DC, dendritic cells; TAM, tumor-associated macrophages; NK, natural killer cells; Treg, regulatory T-cells; Tc, T cells; MDSC, myeloid-derived suppressor cells.

In a top-down approach, as represented by pro-anakoinotic therapies, biomodulatory drug combinations, including agonists of transcriptional cascades, facilitate therapeutically efficacious communications within biological systems, such as tumors (Figure 5) (183). When oncologists follow the top-down treatment approach, implementing a reverse engineering of the tumor system, they guide components of tumor systems via pro-anakoinotic drugs, by regulating and resetting tumor systems toward a growth attenuating state (10, 145). They may start to monitor phenotypical changes in tumor systems right up to and including clinical read outs, utilizing diversified response parameters not routinely monitored in the context of classical targeted therapies. Biomodulatory therapies elaborate and classify systems behavior according to biochemical, cellular and clinical response patterns, a process, which we have highlighted by the definition of communications-related technical terms, such as anakoinosis, master modifiers of tumor tissues and specific context-dependent changes in tumor systems' behavior, differentiation and transdifferentiation processes (Tables 3, 4). Finally, clinical data from the successful application of the same biomodulatory schedules across histologically different metastatic tumor types is suggestive of unique communicative networks among histologically different metastatic cancers.

Anakoinosis induction keeps in mind the holistic communicative context of the tumor system. It aims at uncovering and systematizing an evolutionary tumor pathophysiology via “reverse engineering” of tumor systems on basis of the tumor's available communication repertoire. Therapeutically, anakoinosis may additionally destabilize tumor systems' robustness by altering validity and function of both oncogene- and non-oncogene-addicted targets (149, 184–186). A cascade of operative actions to achieve growth attenuation must realize the communications-level changes in structures, functions and hubs.

Pro-anakoinotic therapies may omit the necessity for temporary complete remission or molecular complete remission, rather aiming at long-term disease stabilization at minimal toxicity (Figure 3) (10). Anakoinosis-induced biological outcomes include tumor cell differentiation or transdifferentiation, implementation of biological memory, apoptosis induction, destabilization of the tumor's robustness (148), combined up-stream and down-stream modulation of signaling pathways with agonistic and antagonistic acting master modifiers, and “normalization” of homeostatic pathways in tumor systems (Tables 3, 4) (187, 188). Thus, anakoinosis therapeutically mimics what Theodosius Dobzhansky paraphrases with, “nothing in biology and evolutionary theory makes sense except in the light of the ability of living matter to communicate, and by doing so, to solve problems,” in our case urgent therapeutic problems (189).

Successful pro-anakoinotic therapy supports the hypothesis that the genetic background does not exclusively determine tumor phenotype and is not the exclusive basis for designing anti-tumor therapy.

The currently available data on communicative reprogramming are barely adequate for outlining how communication lines in tumors are linked and therapeutically modifiable with master modifiers. However, data on pro-anakoinotic therapy schedules justify the introduction of the term anakoinosis (10). Novel models, organoids, proteomics and lipidomics in patient serum, organ on chip platforms may elucidate many phenotypical data on communications networks for rational planning of pro-anakoinotic therapy schedules and for advancing the use of suitable drugs and drug repurposing (10, 190–192). In parallel, novel system states of cellular systems may be uncovered via pro-anakoinotic therapy schedules. Areas of application of pro-anakoinotic therapy schedules are currently not sufficiently explored or exploited.

Either anakoinosis as therapy approach confers the option to keep metastatic and refractory tumors controlled, or it may even induce continuous complete remission. In so doing, anakoinosis therapies demonstrate that metastatic tumor diseases are not exclusively tumor cell-autonomous diseases, i.e., driven by cancer cells only. Also non-tumor-cell-autonomous communicative processes may keep tumor cells in check and are communicatively linked with tumor cells (Figure 5) (16, 23, 193–197).

## Author Contributions

DH, AR, LG, PP, and CG conceived the review and wrote the manuscript. LG and CG supported through interpretation of data for the work. All the authors revised the manuscript critically, approved the final manuscript, and agreed to be accountable for all aspects of the manuscript.

### Conflict of Interest

The authors declare that the research was conducted in the absence of any commercial or financial relationships that could be construed as a potential conflict of interest.
